# Body Composition and Markers of Cardiometabolic Health in Transgender Youth Compared With Cisgender Youth

**DOI:** 10.1210/clinem/dgz029

**Published:** 2019-09-23

**Authors:** Natalie J Nokoff, Sharon L Scarbro, Kerrie L Moreau, Philip Zeitler, Kristen J Nadeau, Elizabeth Juarez-Colunga, Megan M Kelsey

**Affiliations:** 1 Department of Pediatrics, University of Colorado Anschutz Medical Campus; 2 Center for Women’s Health Research, University of Colorado Anschutz Medical Campus; 3 Department of Community and Behavioral Health, University of Colorado School of Public Health; 4 Adult and Child Consortium for Health Outcomes Research and Delivery Science (ACCORDS); 5 Rocky Mountain Prevention Research Center, University of Colorado Anschutz Medical Campus School of Public Health; 6 Department of Medicine, University of Colorado Anschutz Medical Campus; 7 Eastern Colorado Veteran Affairs (VA) Geriatric Research Education and Clinical Center (GRECC); 8 Department of Biostatistics and Informatics, University of Colorado School of Public Health

**Keywords:** transgender, testosterone, estradiol, insulin resistance, body composition

## Abstract

**Context:**

As many as 1.8% of adolescents identify as transgender and many more seek care, yet the impact of gender-affirming hormone therapy (GAHT) on cardiometabolic health is unknown.

**Objective:**

To determine insulin sensitivity and body composition among transgender females (TF) and males (TM) on estradiol or testosterone, compared with cisgender females (CF) and males (CM).

**Design:**

Pilot, cross-sectional study conducted from 2016–2018.

**Setting:**

Academic regional transgender referral center.

**Participants:**

Transgender adolescents on either testosterone or estradiol for at least 3 months were recruited. Nineteen TM were matched to 19 CM and 42 CF on pubertal stage and body mass index (BMI). Eleven TF were matched to 23 CF and 13 TF to 24 CM on age and BMI.

**Main Outcome Measures:**

1/[fasting insulin] and body composition (dual-energy x-ray absorptiometry).

**Results:**

Total body fat was lower in TM than CF mean ± SD: (29% ± 7% vs 33% ± 7%; *P* = 0.002) and higher than in CM (28% ± 7% vs 24% ± 9%; *P* = 0.047). TM had higher lean mass than CF (68% ± 7% vs 64% ± 7%, *P* = 0.002) and lower than CM (69% ± 7% vs 73% ± 8%; *P* = 0.029). Insulin sensitivity was not different between the groups.

TF had lower body fat than CF (31% ± 7% vs 35% ± 8%; *P* = 0.033) and higher than CM (28% ± 6% vs 20% ± 10%; *P* = 0.001). TF had higher lean mass than CF (66% ± 6% vs 62% ± 7%; *P* = 0.032) and lower than CM (69% ± 5% vs 77% ± 9%; *P* = 0.001). TF were more insulin resistant than CM (0.078 ± 0.025 vs 0.142 ± 0.064 mL/μU; *P* = 0.011).

**Conclusions:**

Transgender adolescents on GAHT have significant differences in body composition compared with cisgender controls, with a body composition intermediate between BMI-matched CMs and CFs. These changes in body composition may have consequences for the cardiometabolic health of transgender adolescents.

**ClinicalTrials.gov:**

NCT02550431

In the United States, 0.7% to 1.8% of youth identify as transgender (defined as gender identity that is different or opposite from sex at birth) (1, 2). Some transgender youth will start gender-affirming hormone therapy (GAHT); referrals to centers and providers specializing in this care are rising (3). For patients with a diagnosis of gender dysphoria, a gonadotropin-releasing hormone analogue (GnRHa) may be started at Tanner 2 pubertal development and testosterone or estradiol started later in adolescence or adulthood (4). For transgender women who did not receive early GnRHa, an anti-androgen is typically started along with estradiol (4). Available data in adults, mostly from Europe, show that transgender women treated with estradiol have a higher incidence of strokes and venous thromboembolism (VTE) than both cisgender women and men (those whose gender identity corresponds with sex at birth) (5). Both transgender women treated with estradiol and transgender men treated with testosterone have a higher incidence of myocardial infarction (MI) than cisgender women (5). Furthermore, there are sex differences in heart disease; cisgender men have a higher prevalence of and death rate from heart disease than cisgender women (6). A meta-analysis of markers of cardiometabolic health and risk in transgender adults on GAHT showed changes in lipid parameters for individuals on hormone therapy, but the data on outcomes such as MI, stroke, VTE and death were sparse (7). Another meta-analysis of longitudinal studies of transgender adults on GAHT showed that both transgender men and women gain weight on GAHT. Specifically, transgender women on GAHT have an increase in body fat and a decrease in lean mass, whereas transgender men on GAHT have the opposite (8).

There are many gaps in the literature including: 1) virtually no data on adolescents starting hormone therapy, despite this being a rapidly increasing population with therapy started at younger ages than in the past (4), 2) sparse data from the United States, where different medications are available and where there may be differences in treatment or approach to care and 3) very few studies compare data between transgender and cisgender individuals. The present study addresses these gaps by evaluating markers of cardiometabolic health in US transgender adolescents on hormone therapy compared with cisgender adolescents.

The aims of our cross-sectional pilot study are to evaluate insulin sensitivity and body composition among adolescent transgender females (TF) and males (TM) receiving estradiol or testosterone treatment, respectively, matched to cisgender females (CF) and males (CM) of the same body mass index (BMI) and either age or pubertal stage.

## Materials and Methods

### Participants

Transgender youth up to age 21 years were recruited between 2016 and 2018 from the TRUE Center for Gender Diversity at Children’s Hospital Colorado. Participants were eligible if they had been on either testosterone or estradiol treatment for at least 3 months. Youth were excluded if they had significant medical or psychiatric comorbidities (including diabetes or antipsychotic treatment). The study was approved by the Colorado Multiple Institutional Review Board and consent and/or assent was obtained from all participants and their guardians (for those under age 18 years).

Twenty-one TM and 14 TF participated. The electronic medical records of the transgender participants were reviewed and the start dates for testosterone or estradiol and/or GnRHa treatment and duration of treatment was collected.

Data on healthy cisgender controls were obtained from 2 studies performed at the same institution: the Colorado RESistance to InSulin in Type 1 ANd Type 2 diabetes (RESISTANT) study and the Health Influences in Puberty (HIP) study. Inclusion criteria for RESISTANT have previously been described (9). In RESISTANT, pubertal adolescents ages 12 to 19 years were recruited. Only controls were utilized from RESISTANT. In RESISTANT, youth were included if they were Tanner stage > 1 and sedentary (< 3 hours regular exercise/week) and excluded if they had hypertension, hemoglobin < 9 mg/dL, serum creatinine > 1.5 mg/dL, smoked, had medication-dependent asthma and other conditions that precluded exercise testing or were on medications affecting insulin resistance. In the HIP study, adolescents in early puberty (Tanner Stage 2–3) who were either normal weight or obese were recruited from 2009 to 2015 through local pediatric practices (10). Presence of diabetes, prediabetes, or medications affecting glucose metabolism was exclusionary. Body composition was measured using dual-energy absorptiometry (DXA) in all studies. Participants in each study had a puberty exam performed by a pediatric endocrinologist.

### Research visit

All transgender participants had a research visit in the morning at the University of Colorado Anschutz Medical Center (CU-AMC) pediatric Clinical Translational Research Center (CTRC) after an overnight fast. For participants taking testosterone, the visit was performed prior to their testosterone injection to obtain a trough value. Pubertal staging was performed by a pediatric endocrinologist using the standards of Tanner and Marshall for breast development (using inspection and palpation), testicular development, and pubic hair (11, 12); testicular volume was assessed using a Prader orchidometer and assigned a Tanner stage equivalent as follows: Tanner 1 < 4 mL; Tanner 2 ≥ 4 mL and < 8 mL; Tanner 3 ≥ 8 and < 12 mL; Tanner 4 ≥ 12 and ≤15 mL; Tanner 5 > 15 mL. Several participants declined genital examination. TM who had already undergone chest masculinizing surgery were assigned Tanner 5 breast development for this analysis. Height was measured on a Harpenden stadiometer and weight on a digital electronic scale. Height and weight were recorded to the nearest 0.1 centimeter and kilogram, respectively. Blood pressure was measured, sitting for at least 5 minutes, with an age-appropriate manual cuff. Weight, height, and blood pressure were each measured twice and averaged. BMI was calculated by weight in kilograms divided by height in meters squared. As all participants were younger than 20 years, pediatric norms for BMI were used (percentile, where 5% to < 85% is normal weight, 85% to < 95% is overweight and ≥ 95% is obese). The 2000 CDC Growth Charts were used to calculate percentiles (13).

Participants filled out a demographic questionnaire and all study data were managed using REDCap electronic data capture tools hosted at the CU-AMC (14).

Fasted blood samples were drawn in the morning and body composition was measured by DXA (Discovery A, Hologic).

### Laboratory assays

Serum/plasma fasted blood samples were assayed for glucose, insulin, lipid panel, aspartate aminotransferase (AST), alanine aminotransferase (ALT), hematocrit, hemoglobin A1C, leptin, sex hormone-binding globulin (SHBG), luteinizing hormone (LH), follicle stimulating hormone (FSH), estradiol, and total testosterone. Laboratory assays were performed by the CU-AMC CTRC Core Laboratory and the UC Health Clinical Laboratory. Glucose was measured by enzymatic UV testing (AU480 Chemistry Analyzer, Beckman Coulter), with inter- and intra-assay coefficient of variations (CV) of 1.44% and 0.67%, respectively, and a sensitivity of 10 mg/dL. Insulin was measured by radioimmunoassay (EMD Millipore, Darmstadt, Germany), with inter- and intra-assay CV of 9.8% and 5.2%, respectively, and sensitivity of 3 μU/mL. Leptin was measured by radioimmunoassay (Millipore, Darmstadt, Germany), with inter- and intra-assay CV of 5.8% and 5.9%, respectively, and sensitivity of 0.5 ng/mL.

Testosterone, estradiol, and SHBG were measured by chemiluminescence (Beckman Coulter). Testosterone inter- and intra-assay CV were 5.1% and 2.1%, respectively, and sensitivity was 17 ng/dL; estradiol inter- and intra-assay CV were 8.2% and 4.3%, respectively, with sensitivity of 10.0 pg/mL; SHBG inter- and intra-assay CV were 5.7% and 3.6%, respectively, and sensitivity was 3 nmol/L. In HIP and RESISTANT, testosterone and insulin were measured on the same platforms in the same lab.

Total cholesterol, triglycerides and high-density lipoprotein (HDL) were directly measured and low-density lipoprotein (LDL) was calculated using the Friedewald formula (for units in mg/dL) (15). Insulin sensitivity was estimated by the inverse of the fasting insulin concentration (1/[fasting insulin]), which is correlated with insulin sensitivity measured with a hyperinsulinemic euglycemic clamp (16); lower values for 1/insulin indicate worse insulin sensitivity. Homeostatic model assessment of insulin resistance (HOMA-IR) was calculated as (glucose∙insulin)/405 (with units in mg/dL) (17), with higher values indicating worse insulin sensitivity. Free androgen index (FAI) was calculated as the ratio of total testosterone to SHBG ([testosterone/SHBG] × 100) (18).

### Statistical analysis

TM participants (n = 21, ages 15.1–19.8 years) were matched on body mass index (BMI) percentile to cisgender controls, allowing for a +/- 6% difference using the GREEDY matching algorithm (19). All controls and all but 2 TM were Tanner Stage 5 (missing data for 2 TM participants). TM participants (n = 19) were matched to CM (n = 19, ages 13.1–19.7 years) and CF (n = 42, ages 11.7–18.9 years) with a range of 1 to 3 CF matches per TM case (1:1, n = 19; 1:2, n = 14; 1:3, n = 9). Of the 19 TM matched to CM and CF, 17 were in both comparisons.

TF (n = 14, ages 14.5–19.4 years) were matched (2 were unable to be matched due to lack of matching weight controls) to cisgender youth on age (within a year) and BMI in 2 phases. The first phase performed a one-to-one match using the GREEDY algorithm with BMI percentile +/- 12.5% and age within a year. The second phase identified additional matches with BMI percentile in the same category and age within a year. TF (n = 13) were matched to CM (n = 24, ages 14.5–19.8 years) with a range of 1 to 5 matches per TF case (1:1, n = 8; 1:2, n = 2; 1:3, n = 1; 1:4, n = 1; 1:5, n = 1). TF (n = 11) were matched to 23 CF (ages 14.2–18.3) with a range of 1 to 4 matches per TF case (1:1, n = 5; 1:2, n = 2; 1:3, n = 2; 1:4, n = 2). Ten TF were in both comparisons. One participant did not have estradiol results.

Because the AST and ALT for the cisgender participants were measured by a different assay, a correction factor was applied to the transgender participants: corrected AST = (measured AST + 14.374)/0.8334 and corrected ALT = (measured ALT +10.058)/0.9319. Deming regression was used to build a regression model and determine the correction needed to make the results equivalent using the parameter estimates obtained from the regression.

Tests of differences between TF and controls and TM and controls were performed by running a mixed linear regression model with a random effect for the matched set. Compound symmetry was used for the covariance structure and the restricted maximum likelihood method was used to estimate the covariance parameters. Comparisons with TM were adjusted for age in years because we did not match on age for the TM group. Outcomes that were not normally distributed were log transformed and the log-transformed *P* values are presented. For group comparisons in which there was more than one cisgender match for the transgender participant, the means of the matched set are presented (eg, if 3 CF were matched to 1 TF, the mean of outcome for the 3 CF was obtained; the means and SD of the matched set means are reported).

Our preferred approach would have been to match on pubertal stage and adjust for age for all groups, given that insulin resistance changes with pubertal stage (20). Because all TM participants were Tanner 5, we adjusted for age. However, for the TF cohort, more participants had received a GnRHa and had a lower Tanner stage than similar-age controls, so we were unable to match on Tanner stage. Therefore, we matched on age, to try to account for differences in insulin sensitivity throughout puberty. We did not adjust for pubertal stage, due to missing data.

Spearman correlations were performed to evaluate for correlations between: 1) % body fat and leptin and 2) % lean mass and inverse of fasting insulin. Analyses were conducted using SAS version 9.4 (SAS Institute). *P* values < 0.05 were considered significant. We did not correct for multiple comparisons since this was a pilot study and all findings are considered exploratory.

## Results

Demographics of the overall group are presented in Tables 1 and 2. TM were on an average testosterone dose of 217 ± 88 mg/month for an average treatment duration of 11.2 ± 5.9 months. Twelve (57%) were using intramuscular (IM) injections and 9 (43%) were using subcutaneous (SQ) injections. None were on a GnRHa at the time of the study visit but 1 participant had recently discontinued the GnRHa (length of GnRHa therapy 17.1 months) and therefore may still have been experiencing the effects of the medication. An additional 3 participants had used a GnRHa in the past. Four participants were using a progestin at the time of the study visit (3 on medroxyprogesterone, 1 with an etonogestrel implant). Six participants had undergone chest masculinizing surgery. None had received any other types of surgeries. All TM had previously had menarche (response for 1 missing) and only 2 were currently having menses (Table 1).

**Table 1.** Demographics of Transgender Males and Cisgender Females and Males

	Transgender Male (n = 21)	Cisgender Female (n = 42)	Cisgender Male (n = 19)
Age (years)	17.0 ± 1.4	15.2 ± 1.9	15.3 ± 1.6
Race			
White	15 (71)	28 (67)	14 (74)
Asian	1 (5)	3 (7)	2 (11)
African-American	0	9 (21)	2 (11)
Native American/ Alaska Native	1 (5)	1 (2)	0
More than one race	3 (14)	0	1 (5)
Unknown/not reported	1 (5)	1 (2)	0
Ethnicity			
Hispanic	7 (33)	14 (33)	3 (16)
Not Hispanic	14 (67)	26 (62)	16 (84)
Unknown/not reported	0	2 (5)	0
Pubic hair Tanner stage			
1	0	0	0
2	0	0	0
3	0	0	1 (5)
4	1 (5)	8 (19)	7 (37)
5	14 (67)	6 (14)	9 (47)
Missing	6 (29)	28 (67)	2 (11)
Breast/testicular Tanner stage			
Stage 5	19 (90)	42 (0)	19 (100)
Missing	2 (10)	0	0
Age of menarche	11.9 ± 1.1	12.4 ± 1.4†	---
Family history*			
Hypertension	13 (68)	12 (86)	15 (88)
Hypercholesterolemia	10 (52)	11 (79)	14 (82)
Type 2 diabetes	10 (52)	11 (46)	8 (47)

Values above represent the entire cohort used and are either presented as mean ± SD or n (%). Different TM participants were used to match to different cisgender males (CM) or females (CF) based on the ideal match for BMI. Mean ± SD or n (%). *For CM and CF, percentages are given out of total number of reported values (non-missing). Family history data was missing for several conditions. For CF, there was missing family history of hypertension and hypercholesterolemia for 28 and type 2 diabetes for 18 participants. For CM, there was missing family history of hypertension, hypercholesterolemia and type 2 diabetes for 2 participants. †For CF, age of menarche was missing for 16 participants (38%).

**Table 2.** Demographics of Transgender Females and Cisgender Females and Males

	Transgender Female (n = 14)	Cisgender Female (n = 23)	Cisgender Male (n = 24)
Age (years)	16.3 ± 1.4	15.9 ± 1.4	15.7 ± 1.4
Race			
White	12 (86)	19 (83)	19 (79)
Asian	0	1 (4)	1 (4)
African-American	0	3 (13)	2 (8)
Native American/ Alaska Native	1 (7)	0	0
More than one race	1 (7)	0	0
Unknown/not reported	0	0	2 (8)
Ethnicity			
Hispanic	2 (14)	7 (30)	5 (21)
Not Hispanic	10 (71)	16 (70)	18 (75)
Unknown/not reported	2 (14)	0	1 (4)
Pubic hair Tanner stage			
1	0	0	0
2	1 (7)	0	0
3	0	0	3 (13)
4	1 (7)	5 (21)	7 (29)
5	9 (64)	1 (4)	3 (13)
Missing	3 (21)	17 (74)	11 (46)
Breast/testicular Tanner stage			
Stage 1	1 (7)	0	0
Stage 2	1 (7)	0	0
Stage 3	2 (14)	0	6 (25)
Stage 4	3 (21)	4 (17)	8 (33)
Stage 5	4 (29)	19 (83)	10 (42)
Missing	3 (21)	0	0
Age of menarche	---	12.8 ± 1.6†	---
Family history*			
Hypertension	9 (64)	4 (40)	12 (92)
Hypercholesterolemia	11 (79)	5 (83)	11 (85)
Type 2 diabetes	8 (57)	4 (33)	8 (57)

Values above represent the entire cohort used and are either presented as mean ± SD or n (%). TF participants were used to match to different cisgender males (CM) or females (CF) based on the ideal match for age and BMI. Mean ± SD or n (%). *For CM and CF, percentages are given out of total number of reported values. Family history data was missing for several conditions. For CF, there was missing family history of hypertension for 17, hypercholesterolemia for 17 and diabetes for 11 participants. For CM, there was missing family history of hypertension for 11, hypercholesterolemia for 11, diabetes for 10 participants. †For CF, age of menarche was missing for 9 participants (39%).

TF were taking an average estradiol dose of 1.5 ± 1.0 mg/day with an average treatment duration of 12.3 ± 9.9 months (5 on oral, 9 on sublingual). Four were on a GnRHa at the time of the study visit and a total of 6 had been on a GnRHa in the past. Seven were on spironolactone for androgen blockade and 1 was on IM medroxyprogesterone acetate for puberty suppression.

### Transgender males compared with cisgender females

**Figure 1.**
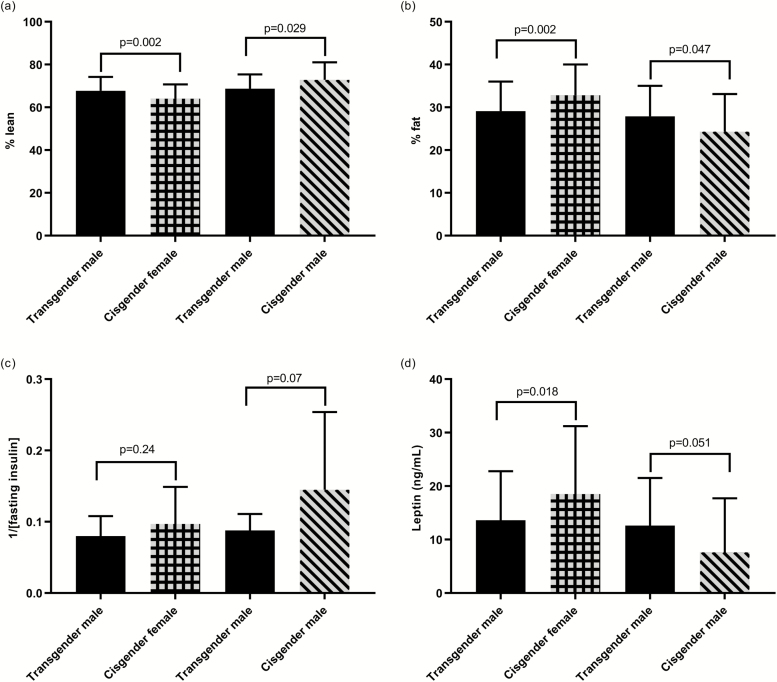
Body composition, insulin sensitivity, and leptin in transgender males and cisgender males and females. Means and SD are presented. Transgender males are presented twice because not all the same individuals are compared with both cisgender males and females (17 are in both comparisons).

Markers of cardiometabolic health and hormone concentrations for TM are displayed in Table 3 and Fig. 1. TM had a higher AST (*P* = 0.001), lower HDL (*P* = 0.043) and lower leptin (*P* = 0.018) than CF. Body composition was significantly different between groups (Table 3, Fig. 1a and b). TM had a lower percent body fat (*P* = 0.002) and fat mass (*P* = 0.029), and higher percent lean tissue (*P* = 0.002) and lean mass (*P* = 0.039) than CF. Compared with CF, TM had higher serum testosterone (*P* < 0.001) and FAI (*P* < 0.001) and lower SHBG (*P* < 0.001). There were no differences in insulin sensitivity between TM and CF.

**Table 3.** Markers of Cardiometabolic Health and Hormone Concentrations for Transgender Males Compared With Cisgender Males and Females

	Transgender Male (n = 19)	Cisgender Female (n = 42†)	Transgender Male (n = 19)	Cisgender Male (n = 19)
Age	16.9 ± 1.4	14.9 ± 1.7	17.1 ± 1.4	15.3 ± 1.6
BMI (%)	71 ± 22	71 ± 21	63 ± 28	64 ± 28
Systolic BP (mm Hg)	108 ± 9	111 ± 8	108 ± 9	115 ± 13**
Diastolic BP (mm Hg)	70 ± 7	66 ± 6	69 ± 8	67 ± 10
Inverse of fasting insulin (mL/μU)	0.080 ± 0.028	0.097 ± 0.052	0.088 ± 0.023	0.145 ± 0.109
HOMA-IR	3.3 ± 2.0	3.2 ± 1.5	2.7 ± 0.8	2.2 ± 1.4
Fasting glucose (mg/dL)	89 ± 5	85 ± 6	88 ± 5	86 ± 10
Hemoglobin A1C (%)	5.3 ± 0.2	5.2 ± 0.2	5.3 ± 0.3	5.3 ± 0.3
AST (U/L)	39 ± 5	29 ± 8**	39 ± 4	36 ± 16
ALT (U/L)	26 ± 5	26 ± 7	25 ± 6	34 ± 17***
Total cholesterol (mg/dL)	147 ± 16	153 ± 29	143 ± 19	146 ± 22
Triglycerides (mg/dL)	75 ± 21	100 ± 45	76 ± 23	91 ± 30
HDL (mg/dL)	40 ± 5	46 ± 7*	41 ± 5	46 ± 9
LDL (mg/dL)	92 ± 16	87 ± 22	87 ± 19	82 ± 19
Total estradiol (pg/mL)	43 ± 23	63 ± 40	46 ± 22	24 ± 11**
Total testosterone (ng/dL)	363 ± 220	39 ± 13***	378 ± 219	445 ± 152
LH (mIU/mL)	3.5 ± 4.8	---	5.3 ± 7.0	---
FSH (mIU/mL)	3.5 ± 3.3	---	4.2 ± 3.4	---
SHBG (nmol/L)	24 ± 11	47 ± 25***	26 ± 11	36 ± 13
Free androgen index	65 ± 47	4 ± 2***	64 ± 47	48 ± 16

Values are given as mean ± SD. Of the 19 transgender males compared with cisgender females and the 19 compared with cisgender males, 17 are in both comparisons. Abbreviations: BP, blood pressure; FSH, follicle stimulating hormone; LH, luteinizing hormone. †means and standard deviations are reported for the 19 matched sets for cisgender females, *P < 0.05, **P ≤ 0.01, ***P ≤ 0.001 (*P* values represent significance from log-transformed variables when relevant)

### Transgender males compared with cisgender males

Compared with CM, TM had a lower systolic blood pressure (*P* = 0.005) and ALT (*P* < 0.001). Body composition was significantly different between groups (Table 3, Fig. 1a and b). TM had a higher percent body fat (*P* = 0.047), and lower percent lean tissue (*P* = 0.029) and lean mass (*P* < 0.001) than CM. Compared with CM, TM had higher serum estradiol (*P* = 0.004). There were no differences in insulin sensitivity between TM and CM.

### Transgender females compared with cisgender females

**Figure 2.**
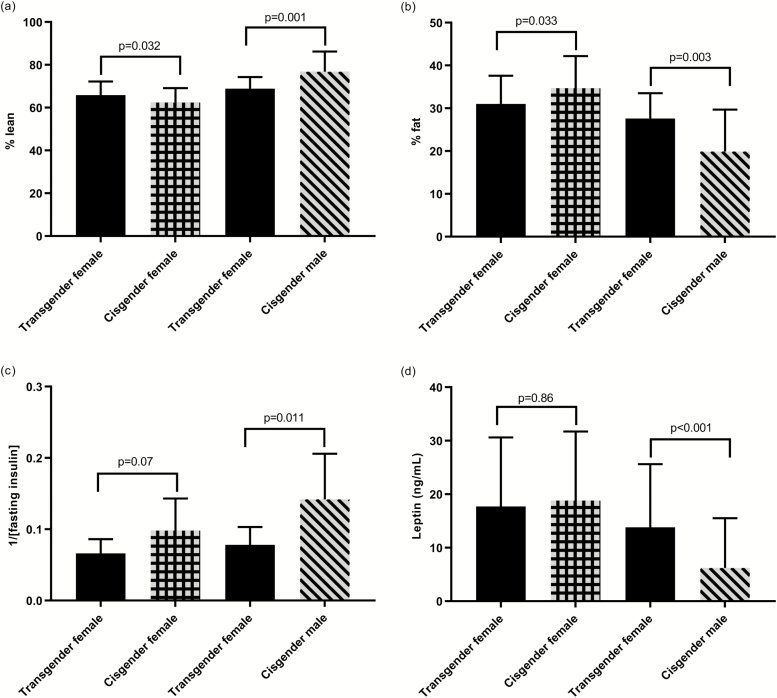
Body composition, insulin sensitivity, and leptin in transgender females and cisgender males and females. Means and SD are presented. Transgender females are presented twice because not all the same individuals are compared with both cisgender males and females (10 are in both comparisons).

Markers of cardiometabolic health and hormone concentrations for TF are shown in Table 4 and Fig. 2. TF had a higher AST than CF (*P* < 0.001). Body composition was significantly different between groups (Table 4, Fig. 2a and b). Compared with CF, TF had lower percent body fat (*P* = 0.033) and higher percent lean tissue (*P* = 0.032), lean mass (*P* = 0.004), total testosterone (*P* < 0.001), and FAI (*P* = 0.002) than CF. There were no differences in insulin sensitivity between TF and CF. Results were then stratified by spironolactone use (yes/no, data not shown). The mean systolic blood pressure was lower in those on spironolactone compared with those not on spironolactone. The means for the inverse of fasting insulin, AST, percent body fat, percent lean tissue, lean mass, total testosterone, and FAI were all in the same direction as those in Table 4 and Fig. 2 for TF compared with CF.

**Table 4.** Markers of Cardiometabolic Health and Hormone Concentrations Transgender Females Compared With Cisgender Females and Males

	Transgender Female (n = 11)	Cisgender Female (n = 23†)	Transgender Female (n = 13)	Cisgender Male (n = 24†)
Age	16.2 ± 1.2	16.0 ± 1.3	16.2 ± 1.4	16.1 ± 1.6
BMI (%)	55 ± 34	58 ± 30	46 ± 37	45 ± 36
Systolic BP (mm Hg)	107 ± 12	113 ± 7	106 ± 11	116 ± 8**
Diastolic BP (mm Hg)	70 ± 7	66 ± 7	70 ± 6	67 ± 5
Inverse of fasting insulin (mL/μU)	0.066 ± 0.02	0.098 ± 0.045	0.078 ± 0.025	0.142 ± 0.064*
HOMA-IR	3.8 ± 2.1	2.8 ± 1.4	3.4 ± 2.2	2.1 ± 1.2*
Fasting glucose (mg/dL)	89 ± 5	82 ± 12	90 ± 4	86 ± 6
Hemoglobin A1C (%)	5.2 ± 0.4	5.0 ± 0.2	5.2 ± 0.4	5.1 ± 0.3
AST (U/L)	37 ± 4	23 ± 6***	37 ± 4	34 ± 18
ALT (U/L)	25 ± 5	26 ± 6	24 ± 5	32 ± 21
Total cholesterol (mg/dL)	148 ± 23	145 ± 20	152 ± 22	136 ± 25
Triglycerides (mg/dL)	77 ± 34	74 ± 21	81 ± 34	97 ± 30
HDL (mg/dL)	49 ± 10	46 ± 10	50 ± 10	43 ± 6*
LDL (mg/dL)	83 ± 20	84 ± 20	85 ± 20	74 ± 22
Total estradiol (pg/mL)	98 ± 135	96 ± 127	124 ± 162	23 ± 9**
Total testosterone (ng/dL)	224 ± 182	43 ± 10***	252 ± 214	412 ± 168*
LH (mIU/mL)	3.6 ± 3.2	---	3.5 ± 2.9	---
FSH (mIU/mL)	2.1 ± 1.9	---	1.9 ± 1.8	---
SHBG (nmol/L)	49 ± 36	50 ± 30	50 ± 48	40 ± 16
Free androgen index	33 ± 36	5 ± 3**	36 ± 34	37 ± 16

Values are given as mean ± SD. Of the 11 transgender females compared with cisgender females and the 13 compared with cisgender males, 10 are in both comparisons. B*P* = blood pressure, LH = luteinizing hormone, FSH = follicle stimulating hormone, †means and standard deviations are reported for the 11 matched sets for cisgender females and 13 matched sets for cisgender males, *P < 0.05, **P ≤ 0.01, ***P ≤ 0.001 (p-values represent significance from log-transformed variables when relevant)

### Transgender females compared with cisgender males

TF were more insulin resistant than CM, with a lower inverse of fasting insulin (*P* = 0.011) and a higher HOMA-IR (*P* = 0.012). TF had a lower systolic blood pressure (*P* = 0.007), and higher HDL (*P* = 0.023) and leptin (*P* < 0.001) than CM. Body composition was significantly different between groups (Table 4, Fig. 2a and b). Compared with CM, TF had higher percent body fat (*P* = 0.003) and fat mass (*P* = 0.004) and lower percent lean tissue (*P* = 0.001). TF had higher estradiol (*P* = 0.005) and lower total testosterone (*P* = 0.012) than CM. Results were then stratified by spironolactone use (yes/no, data not shown). The mean systolic blood pressure was lower in those on spironolactone compared with those not on spironolactone and this seemed to account for the overall group difference seen. The means for the inverse of fasting insulin, HDL, leptin, percent body fat, fat mass, percent lean, total estradiol, and total testosterone were all in the same direction as those in Table 4 and Fig. 2 for TF and CM.

### Correlations and other observations

In the pooled population of TM and cisgender controls, the inverse of fasting insulin correlated with percent lean mass (*r* = 0.40 [95% CI, 0.19-0.57]; *P* = 0.0004) as well as for TF and controls (*r* = 0.65 [95% CI, 0.46-0.79]; *P* < 0.0001). As expected, leptin correlated strongly with percent fat mass for both TM and their controls (*r* = 0.90 [95% CI, 0.84-0.94]; *P* < 0.0001) and TF and their controls (*r* = 0.94 [95% CI, 0.89-0.97]; *P* < 0.0001).

The percentage lean and percentage fat mass were remarkably similar for the TF and TM; these groups were not matched on age or BMI or compared with one another.

## Discussion

The present study demonstrates novel observations with regards to cardiometabolic health parameters in transgender youth treated with GAHT. First, both TM and TF had a body composition (defined by percent fat and lean on DXA) that is intermediate between CF and CM. Second, there were both favorable and unfavorable changes in markers of cardiometabolic health for TF and TM compared with BMI-matched cisgender youth. The only group that had a difference in insulin sensitivity were the TF compared with CM, with TF being less insulin sensitive than CM.

Most participants in this study had not received a GnRHa and went through most or all their endogenous puberty before starting testosterone or estradiol. Body composition in the TM may be explained by a female pattern of pubertal fat accrual (21, 22), followed by gains in lean mass with testosterone, a known anabolic agent (23). Differences in body composition in the TF may be explained by a male pattern of lean mass accrual during puberty (22), followed by a gain in percent fat and concomitant rise in leptin due to estradiol treatment.

The body composition findings are similar to those observed in adults. In a meta-analysis of longitudinal studies of transgender adults treated with GAHT for a duration of 3 to 24 months, all groups had an increase in body weight. TF experienced an increase in body fat and decrease in lean body mass, whereas TM experienced the opposite changes (8). Participants were on a variety of hormone regimens, some not routinely used in the United States, and most individuals had not received a GnRHa. However, in a multicenter retrospective study of adolescents and young adults, only TM had an increase in BMI on testosterone, whereas there were no changes in BMI for TF after starting estradiol (24). In our cross-sectional study, TM and TF had an intermediate body composition, with percent fat and lean mass between CM and CF values. Similarly, leptin, a hormone secreted from fat cells, was higher in TF compared with CM and lower in TM than CF.

Some studies that have examined insulin sensitivity for adults on GAHT have shown decreased insulin sensitivity for both TF and TM and an increase in fasting insulin for TF (25). Another study showed that both TM and TF on GAHT were more insulin resistant after 4 months of hormone therapy compared with baseline, measured by hyperinsulinemic euglycemic clamp, the gold standard for measuring insulin sensitivity (26). However, TF in that study were treated with ethinyl estradiol (rather than 17β-estradiol, as used in the current study), which is known to have an adverse effect on glucose and insulin (27). Our study was a cross-sectional comparison with control populations, rather than an intra-individual comparison before and after starting treatment, and the TF were more insulin resistant than CM but not CF. In Belgium, both TM and TF have a higher prevalence of type 2 diabetes than CM or CF (28), although the same has not been demonstrated in the United States. (29).

Many of the studies on cardiometabolic health in transgender individuals involve small cohorts, followed longitudinally. However, it is also important to compare outcomes to appropriately matched cisgender participants, as there are known sex differences in cardiometabolic health that begin to emerge in puberty. Youth become more insulin resistant in puberty (beginning at Tanner 2, peaking at Tanner 3, and returning to near prepubertal values sometime after puberty is completed) (20). There are also sex differences in pubertal insulin resistance, with cisgender girls being more insulin resistant than boys; while insulin resistance is strongly related to BMI and body fat, these do not entirely account for the sex differences seen (20). Furthermore, type 2 diabetes in youth is more common among cisgender girls than boys (30, 31), a sex difference not seen in adults (32). In adults, worse insulin resistance is associated with incident coronary heart disease, which has been shown to be more common among transgender than cisgender adults (5, 33). The impact of GnRHa, testosterone, and estradiol started during puberty on present insulin resistance and future risk of type 2 diabetes and heart disease warrants further study.

In TM adults, testosterone therapy is associated with increased LDL and triglycerides and decreased HDL (7). A retrospective study in adolescents and young adults also found that TM on testosterone had a decrease in HDL (24). We found that TM adolescents had lower HDL than CF but no other differences in lipids and no differences compared with CM. TF adults on estradiol had an increase in triglycerides (7). We did not find any statistically significant differences in triglycerides between TF and CM or CF, but TF did have higher HDL than CM, which is an expected effect of estradiol (34).

As many more transgender youth seek GAHT, it is important to have a better understanding of the impact, not only of testosterone and estradiol, but also of GnRH analogues, on short- and long-term cardiometabolic health. The current study, while cross-sectional, has many strengths. There have been very few rigorously performed studies in transgender youth. One published study, although multicenter, was retrospective, with labs performed at different locations and not necessarily fasting (24). Additionally, although body composition has been measured in transgender adults, there are limited available data in transgender adolescents (35). And while most studies in transgender adults have been longitudinal, very few have employed a comparison group with similar characteristics and BMI. There were also several limitations to our study. It was a cross-sectional study, rather than longitudinal, so we do not know about changes before and after GAHT. Some participant data were excluded because there were no available matches. The sample size is small and some participants were on a GnRHa and others were not (and the numbers were too small to identify differences between these 2 groups). Testosterone was not measured by mass spectroscopy in any of the studies. However, sex steroid concentrations were not the primary outcome of this study and were not used in any correlations or outcomes related to body composition or insulin sensitivity. Lastly, the results may not necessarily reflect the impact of exogenous testosterone or estradiol in isolation as several participants were on an additional medication (4 TM on a progestin, 4 TF on GnRHa, 7 TF on spironolactone, 1 TF on a progestin). Spironolactone, a mineralocorticoid receptor antagonist, is an antihypertensive agent (36), improves insulin sensitivity (37), and results in favorable changes in markers of cardiometabolic risk in animal (38) and human (39) studies. Our cohort was not powered to evaluate differences between those on spironolactone compared with those not on spironolactone, although future studies should investigate the optimal means of testosterone suppression for TF on markers of cardiometabolic health. However, since GAHT often includes combinations of sex steroids and other medications, understanding the risks and benefits of these combinations, in addition to the individual components is important.

In conclusion, we show that among transgender adolescents using GAHT for approximately 1 year, there are significant differences in body composition between transgender and cisgender adolescents, with transgender adolescents having a body composition intermediate between cisgender adolescents of the same BMI. There were also differences in markers of cardiometabolic health between transgender and cisgender youth, the most notable being that TF participants were more insulin resistant than CM. Based on the results of this pilot study, further exploration is needed to understand the impact of starting testosterone or estradiol treatment in adolescence, with or without prior pubertal blockade, on short- and long-term cardiometabolic health.

## Abbreviations

ALT: alanine aminotransferase
AST: aspartate aminotransferase
BMI: body mass index
CF: cisgender female
CM: cisgender male
CU-AMC: University of Colorado Anschutz Medical Center
CTRC: Clinical Translational Research Center
DXA: dual-energy x-ray absorptiometry
FAI: free androgen index
FSH: follicle stimulating hormone
GAHT: gender-affirming hormone therapy
GnRHa: gonadotropin-releasing hormone analogue
HDL: high-density lipoprotein
HOMA-IR: homeostatic model assessment of insulin resistance
IM: intramuscular
LDL: low-density lipoprotein
LH: luteinizing hormone
SHBG: sex hormone-binding globulin
SQ: subcutaneous
TF: transgender female
TM: transgender male
VTE: venous thromboembolism.

## Additional Information

***Disclosure Summary:*** NN has previously consulted for Antares Pharma, Inc.

***Data Availability:*** The datasets generated during and/or analyzed during the current study are not publicly available but are available from the corresponding author on reasonable request.
